# Supplementary material for: Hot spot prediction in protein-protein interactions by an ensemble system
Source: BMC Syst Biol. 2018 Dec 31;12(Suppl 9):132. doi: 10.1186/s12918-018-0665-8 (PMC6311905; doi:10.1186/s12918-018-0665-8)
Supplement: Supplementary file 2 — All relevant MATLAB code. A simple Matlab implement of our predictor. (ZIP 19,742 kb) [file 12918_2018_665_MOESM2_ESM.zip › Additional file 2/Read ASA/ASA website/SKEMPI/1BRSA.htm]

 NetSurfP - Protein Surface Accessibility and Secondary Structure Predictions

  

|  |  |  |
| --- | --- | --- |
|  |  | NetSurfP - Protein Surface Accessibility and Secondary Structure Predictions  Technical University of Denmark |

  


---

```
# For publication of results, please cite:
# A generic method for assignment of reliability scores applied to solvent accessibility predictions.
# Bent Petersen, Thomas Nordahl Petersen, Pernille Andersen, Morten Nielsen and Claus Lundegaard
# BMC Structural Biology 2009, 9:51 doi:10.1186/1472-6807-9-51
#
# Column 1: Class assignment - B for buried or E for Exposed - Threshold: 25% exposure, but not based on RSA
# Column 2: Amino acid
# Column 3: Sequence name
# Column 4: Amino acid number
# Column 5: Relative Surface Accessibility - RSA
# Column 6: Absolute Surface Accessibility
# Column 7: Z-fit score for RSA prediction
# Column 8: Probability for Alpha-Helix
# Column 9: Probability for Beta-strand
# Column 10: Probability for Coil
E V  1BRSA                  1    0.708 108.835  -0.147   0.016   0.005   0.979
E I  1BRSA                  2    0.367  67.858  -0.366   0.268   0.043   0.689
E N  1BRSA                  3    0.576  84.268  -0.219   0.455   0.046   0.498
E T  1BRSA                  4    0.505  70.099   0.246   0.622   0.015   0.363
B F  1BRSA                  5    0.222  44.455  -0.415   0.782   0.003   0.216
E D  1BRSA                  6    0.495  71.344   0.071   0.782   0.003   0.216
E G  1BRSA                  7    0.469  36.895   0.481   0.782   0.003   0.216
B V  1BRSA                  8    0.041   6.348   0.255   0.923   0.002   0.076
B A  1BRSA                  9    0.071   7.857  -0.084   0.970   0.001   0.030
E D  1BRSA                 10    0.502  72.309   1.212   0.970   0.001   0.030
B Y  1BRSA                 11    0.137  29.256   0.776   0.970   0.001   0.030
B L  1BRSA                 12    0.025   4.596   0.249   0.970   0.001   0.030
E Q  1BRSA                 13    0.363  64.778   0.926   0.923   0.002   0.076
E T  1BRSA                 14    0.674  93.498   0.526   0.858   0.002   0.139
E Y  1BRSA                 15    0.299  63.939   0.157   0.502   0.002   0.495
E H  1BRSA                 16    0.505  91.896  -0.729   0.109   0.005   0.886
E K  1BRSA                 17    0.476  97.831   0.448   0.053   0.005   0.942
B L  1BRSA                 18    0.104  19.006   0.569   0.016   0.005   0.979
B P  1BRSA                 19    0.226  32.027  -0.063   0.016   0.005   0.979
E D  1BRSA                 20    0.535  77.151  -1.096   0.115   0.016   0.868
E N  1BRSA                 21    0.470  68.793  -0.546   0.191   0.086   0.723
B Y  1BRSA                 22    0.251  53.617   0.036   0.307   0.165   0.527
B I  1BRSA                 23    0.078  14.393  -0.032   0.216   0.235   0.548
E T  1BRSA                 24    0.329  45.605   0.535   0.199   0.152   0.649
E K  1BRSA                 25    0.418  86.024   0.952   0.879   0.010   0.111
E S  1BRSA                 26    0.619  72.547   1.068   0.923   0.002   0.076
E E  1BRSA                 27    0.363  63.469   0.829   0.970   0.001   0.030
B A  1BRSA                 28    0.064   7.020  -0.860   0.970   0.001   0.030
E Q  1BRSA                 29    0.525  93.819   1.213   0.923   0.002   0.076
E A  1BRSA                 30    0.676  74.495   0.893   0.858   0.002   0.139
E L  1BRSA                 31    0.274  50.078   0.780   0.694   0.003   0.303
E G  1BRSA                 32    0.478  37.603  -1.503   0.246   0.004   0.750
B W  1BRSA                 33    0.182  43.819  -0.751   0.115   0.016   0.868
E V  1BRSA                 34    0.547  84.089   0.835   0.058   0.017   0.925
E A  1BRSA                 35    0.381  42.030  -1.547   0.018   0.019   0.964
E S  1BRSA                 36    0.539  63.147  -1.126   0.018   0.019   0.964
E K  1BRSA                 37    0.571 117.352  -0.596   0.058   0.017   0.925
B G  1BRSA                 38    0.301  23.697  -1.659   0.058   0.017   0.925
E N  1BRSA                 39    0.437  64.035  -0.708   0.058   0.017   0.925
B L  1BRSA                 40    0.166  30.321  -1.435   0.113   0.043   0.844
E A  1BRSA                 41    0.301  33.148  -0.964   0.184   0.043   0.773
E D  1BRSA                 42    0.625  90.091   0.130   0.191   0.086   0.723
B V  1BRSA                 43    0.248  38.041  -0.745   0.113   0.087   0.800
B A  1BRSA                 44    0.162  17.841   0.002   0.053   0.043   0.903
E P  1BRSA                 45    0.565  80.145  -0.600   0.018   0.019   0.964
E G  1BRSA                 46    0.573  45.119  -2.162   0.018   0.019   0.964
E K  1BRSA                 47    0.525 108.095  -0.443   0.018   0.088   0.893
E S  1BRSA                 48    0.417  48.896  -1.299   0.020   0.205   0.775
B I  1BRSA                 49    0.266  49.228  -0.984   0.020   0.205   0.775
E G  1BRSA                 50    0.456  35.919  -1.477   0.019   0.141   0.840
B G  1BRSA                 51    0.208  16.377  -1.912   0.056   0.142   0.802
E D  1BRSA                 52    0.353  50.838  -0.713   0.021   0.451   0.528
E I  1BRSA                 53    0.299  55.370   0.958   0.023   0.655   0.322
B F  1BRSA                 54    0.105  21.033   0.276   0.022   0.552   0.426
E S  1BRSA                 55    0.353  41.383   0.016   0.022   0.359   0.619
B N  1BRSA                 56    0.183  26.777  -1.122   0.064   0.216   0.721
E R  1BRSA                 57    0.413  94.669  -0.331   0.064   0.216   0.721
E E  1BRSA                 58    0.449  78.423  -0.966   0.056   0.142   0.802
E G  1BRSA                 59    0.444  34.959  -0.522   0.056   0.142   0.802
E K  1BRSA                 60    0.363  74.751  -0.026   0.056   0.142   0.802
B L  1BRSA                 61    0.151  27.666   0.613   0.052   0.084   0.864
B P  1BRSA                 62    0.148  21.030  -0.755   0.053   0.043   0.903
E G  1BRSA                 63    0.631  49.668   0.497   0.018   0.019   0.964
E K  1BRSA                 64    0.479  98.592   0.355   0.018   0.019   0.964
E S  1BRSA                 65    0.736  86.212  -0.122   0.018   0.019   0.964
E G  1BRSA                 66    0.639  50.274  -0.878   0.005   0.015   0.979
E R  1BRSA                 67    0.350  80.219  -0.639   0.004   0.138   0.858
E T  1BRSA                 68    0.302  41.874   1.393   0.004   0.616   0.381
B W  1BRSA                 69    0.116  27.850   0.566   0.002   0.816   0.182
B R  1BRSA                 70    0.187  42.731   1.452   0.001   0.900   0.099
B E  1BRSA                 71    0.217  37.875  -0.672   0.001   0.900   0.099
B A  1BRSA                 72    0.084   9.224   0.996   0.002   0.816   0.182
E D  1BRSA                 73    0.283  40.809   0.692   0.002   0.816   0.182
B I  1BRSA                 74    0.062  11.526   0.704   0.004   0.616   0.381
E N  1BRSA                 75    0.576  84.297   0.318   0.005   0.336   0.660
B Y  1BRSA                 76    0.192  40.988   0.642   0.018   0.088   0.893
E T  1BRSA                 77    0.575  79.808  -0.622   0.018   0.047   0.935
E S  1BRSA                 78    0.529  62.046  -1.483   0.018   0.019   0.964
E G  1BRSA                 79    0.474  37.296  -1.492   0.018   0.047   0.935
E F  1BRSA                 80    0.512 102.698  -1.019   0.018   0.088   0.893
E R  1BRSA                 81    0.293  67.051  -0.270   0.018   0.088   0.893
E N  1BRSA                 82    0.314  45.911  -0.798   0.018   0.047   0.935
B S  1BRSA                 83    0.133  15.634  -1.338   0.004   0.138   0.858
E D  1BRSA                 84    0.314  45.190   0.347   0.004   0.420   0.576
B R  1BRSA                 85    0.085  19.442   0.706   0.002   0.816   0.182
B I  1BRSA                 86    0.057  10.508   0.105   0.001   0.900   0.099
B L  1BRSA                 87    0.036   6.573  -0.397   0.001   0.900   0.099
B Y  1BRSA                 88    0.105  22.332   0.058   0.001   0.900   0.099
B S  1BRSA                 89    0.084   9.892  -1.617   0.004   0.420   0.576
E S  1BRSA                 90    0.553  64.788  -1.039   0.005   0.015   0.979
E D  1BRSA                 91    0.566  81.489  -1.548   0.018   0.019   0.964
E W  1BRSA                 92    0.319  76.792  -0.632   0.020   0.205   0.775
B L  1BRSA                 93    0.205  37.517  -0.085   0.023   0.655   0.322
B I  1BRSA                 94    0.157  29.082   1.298   0.001   0.900   0.099
B Y  1BRSA                 95    0.093  19.960   0.709   0.001   0.900   0.099
B K  1BRSA                 96    0.173  35.545   0.825   0.001   0.900   0.099
B T  1BRSA                 97    0.074  10.250  -0.062   0.004   0.616   0.381
E T  1BRSA                 98    0.406  56.326  -0.050   0.191   0.086   0.723
E D  1BRSA                 99    0.461  66.372  -0.120   0.339   0.016   0.645
B H  1BRSA                100    0.142  25.848   0.536   0.622   0.015   0.363
B Y  1BRSA                101    0.149  31.820   0.670   0.622   0.015   0.363
E Q  1BRSA                102    0.546  97.426   1.417   0.660   0.049   0.291
B T  1BRSA                103    0.237  32.816   0.551   0.660   0.049   0.291
B F  1BRSA                104    0.071  14.330  -0.475   0.701   0.107   0.192
E T  1BRSA                105    0.471  65.272   1.605   0.649   0.163   0.188
E K  1BRSA                106    0.533 109.679   1.444   0.428   0.171   0.402
B I  1BRSA                107    0.223  41.181  -0.405   0.278   0.093   0.628
E R  1BRSA                108    0.756 173.170   0.582   0.016   0.005   0.979

Explain the output.  Go back.
```

---
